# Long-term results and GvHD after prophylactic and preemptive donor lymphocyte infusion after allogeneic stem cell transplantation for acute leukemia

**DOI:** 10.1038/s41409-021-01515-3

**Published:** 2021-11-08

**Authors:** Christoph Schmid, Myriam Labopin, Nicolaas Schaap, Hendrik Veelken, Arne Brecht, Michael Stadler, Juergen Finke, Frederic Baron, Matthew Collin, Gesine Bug, Per Ljungman, Didier Blaise, Johanna Tischer, Adrian Bloor, Aleksander Kulagin, Sebastian Giebel, Norbert-Claude Gorin, Jordi Esteve, Fabio Ciceri, Bipin Savani, Arnon Nagler, Mohamad Mohty

**Affiliations:** 1 Department of Hematology and Oncology, Augsburg University Hospital and Medical Faculty, Augsburg, Germany; 2 EBMT Study Office, Saint Antoine Hospital, Paris, France; 3 INSERM UMR 938, Sorbonne University, Paris, France; 4 Radboud University Medical Centre Nijmegen, Nijmegen, The Netherlands; 5 Department of Hematology, Leiden University Medical Center, Leiden, The Netherlands; 6 Helios Dr. Horst Schmidt Kliniken, Wiesbaden, Germany; 7 Department of Hematology, Hemostasis, Oncology, and Stem Cell Transplantation, Hannover Medical School, Hannover, Germany; 8 Department of Hematology and Medical Oncology, University of Freiburg, Freiburg, Germany; 9 Department of Medicine, Division of Hematology, University of Liège, Belgium Liege,; 10 Bone Marrow Transplant Unit, Northern Centre for Bone Marrow Transplantation, Newcastle-upon-Tyne, UK; 11 Department of Medicine 2, Goethe University Frankfurt, Frankfurt am Main, Germany; 12 Department of Cellular Therapy and Allogeneic Stem Cell Transplantation, Karolinska University Hospital Huddinge, Stockholm, Sweden; 13 Programme de Transplantation & Therapie Cellulaire-Centre de Recherche en Cancérologie de Marseille-Institut Paoli Calmettes, Marseille, France; 14 Department of Medicine 3, Hematology and Oncology, Ludwig-Maximilian-University, Munich, Germany; 15 Stem Cell Transplantation Unit, The Christie NHS Foundation Trust, Manchester, UK; 16 RM Gorbacheva Research Institute, Pavlov University, St. Petersburg, Russia; 17 Department of Bone Marrow Transplantation and Onco-Hematology, Maria Sklodowska-Curie Institute – Oncology Center, Gliwice Branch, Gliwice, Poland; 18 Faculté de Médicine Saint-Antoine and EBM study office, Saint Antoine Hospital, Paris, France; 19 Hospital Clinic Barcelona, Institute of Hematology and Oncology, Barcelona, Spain; 20 Hematology and Bone Marrow Transplantation Unit, San Raffaele Scientific Institute, Milano, Italy; 21 Vanderbilt University Medical Center, Nashville, TN USA; 22 BMT and Cord Blood Bank, Chaim Sheba Medical Center, Tel Aviv University, Tel-Hashomer, Israel; 23 Service d’Hématologie Clinique et Thérapie Cellulaire, Hôpital Saint-Antoine, AP-HP, Sorbonne University, Paris, France

**Keywords:** Cancer immunotherapy, Immunotherapy, Cancer

## Abstract

We report on 318 patients with acute leukemia, receiving donor lymphocyte infusion (DLI) in complete hematologic remission (CHR) after allogeneic stem cell transplantation (alloSCT). DLI were applied preemptively (preDLI) for minimal residual disease (MRD, *n* = 23) or mixed chimerism (MC, *n* = 169), or as prophylaxis in high-risk patients with complete chimerism and molecular remission (proDLI, *n* = 126). Median interval from alloSCT to DLI1 was 176 days, median follow-up was 7.0 years. Five-year cumulative relapse incidence (CRI), non-relapse mortality (NRM), leukemia-free and overall survival (LFS/OS) of the entire cohort were 29.1%, 12.7%, 58.2%, and 64.3%. Cumulative incidences of acute graft-versus-host disease (aGvHD) grade II–IV°/chronic GvHD were 11.9%/31%. Nineteen patients (6%) died from DLI-induced GvHD. Age ≥60 years (*p* = 0.046), advanced stage at transplantation (*p* = 0.003), shorter interval from transplantation (*p* = 0.018), and prior aGvHD ≥II° (*p* = 0.036) were risk factors for DLI-induced GvHD. GvHD did not influence CRI, but was associated with NRM and lower LFS/OS. Efficacy of preDLI was demonstrated by decreasing MRD/increasing blood counts in 71%, and increasing chimerism in 70%. Five-year OS after preDLI for MRD/MC was 51%/68% among responders, and 37% among non-responders. The study describes response and outcome of DLI in CHR and helps to identify candidates without increased risk of severe GvHD.

## Introduction

The graft-versus-leukemia (GvL) effect is the therapeutic cornerstone of allogeneic stem cell transplantation (alloSCT) in acute leukemia (AL) [1]. The infusion of donor lymphocytes (DLI) to patients with established donor chimerism was initially applied in patients with hematological relapse to reinforce GvL reaction [2, 3]. However, even in chronic myelogenous leukemia, results depended on the status of the disease at time of DLI, with patients in molecular relapse showing superior results to those in hematological relapse or blast crisis [4]. Furthermore, DLI was of limited value in the treatment of overt hematological relapse of AL [1, 5, 6]. These experiences prompted clinicians to exploit the GvL reaction in a less proliferative stage, i.e., by giving DLI to patients in complete hematological remission (CHR). These patients were either at high risk of relapse because of T-cell depletion (TCD) of the graft, unfavorable genetics of the leukemia, or advanced disease at SCT; this approach has been referred to as adjuvant or prophylactic DLI (proDLI). Similarly, DLI was given to patients who showed early signs of relapse such as persisting minimal residual disease (MRD), reappearance of molecular markers of the leukemia, or mixed chimerism (MC), which has been referred to as preemptive DLI (preDLI) [7, 8]. Data from various studies suggest that DLI in CHR may have a role in the prevention of AL relapse; however, no systematic analysis of this strategy is available so far, neither concerning the optimal schedule, nor with respect to safety and clinical efficacy [9]. Hence, the Acute Leukemia Working Party (ALWP) of the European Society for Blood and Marrow Transplantation (EBMT) performed a registry-based survey on 318 patients with AL, who received DLI in CHR after alloSCT.

## Patients and methods

Patients were selected from the EBMT registry. The EBMT is a non-profit, scientific society representing >600 transplant centers that are required to report all consecutive stem cell transplantations including annual follow-up. Data are managed in a central database with internet access. Annual audits are performed to verify data accuracy. Patients provide informed consent authorizing the use of their personal information for research purposes before transplantation.

The study was approved by the general assembly of the ALWP. Eligibility criteria were: (1) age ≥18 years, (2) alloSCT from either a matched sibling donor (MSD) or matched unrelated donor (MUD), (3) documented CHR post transplant, (4) ≥1 DLI applied in CHR, i.e., before date of leukemia relapse or last follow-up (LFU), and (5) available information on the reason for application of DLI. Patients receiving any additional antileukemic treatment between SCT and date of relapse or LFU, such as tyrosine kinase inhibitors (TKI), hypomethylating agents (HMA), or conventional chemotherapy, were excluded, as were patients who had received their first DLI after the date of documented relapse, i.e., in a therapeutic setting. A specific questionnaire was distributed among contributing centers to collect information on DLI and graft-versus-host disease (GvHD) post DLI.

### Definitions

PreDLI and proDLI were defined as described [7]. The preDLI cohort could include patients reported to have MC in addition to MRD. Cytogenetics [10, 11], GvHD [12, 13], CHR before SCT [14], relapse, and intensity of conditioning [15, 16] were defined as published. After SCT, complete reconstitution of hematopoiesis was not required for the diagnosis of CHR. Full donor chimerism was defined by the absence of any detectable recipient signal in blood or bone marrow, as indicated by the respective center report. Considering the genetic heterogeneity of AL, MRD measurement was performed according to local standards, using cytogenetics, molecular genetics, or flow cytometry [7, 17, 18]. Following relapse, all deaths were regarded as disease-related, whereas non-relapse mortality (NRM) was defined as death without evidence of relapse or progression of the leukemia. Overall survival (OS) was defined as interval from date of first DLI (DLI1) to date of LFU or date of death, regardless of cause. Leukemia-free survival (LFS) was calculated between the date of DLI1 and relapse, death, or LFU. Response after preemptive DLI was defined by increasing donor chimerism, decreasing MRD load, or improvement of peripheral blood counts, as indicated by the reporting centers.

### Statistics

Outcome variables of interest were response, cumulative relapse incidence (CRI), NRM, acute and chronic GvHD (aGvHD and cGvHD), and OS/LFS. Probabilities of OS and LFS were estimated by the Kaplan–Meier method [19]. Cumulative incidence functions were used to estimate RI and NRM in a competing risk setting. Death and relapse were considered as competing events for GvHD. Results were expressed as the hazard ratio (HR) with a 95% confidence interval (95% CI). All tests were two-sided with the type I error rate fixed at 0.05. For a risk factor analysis of GvHD after DLI, we selected patients free of immunosuppressive treatment and without acute GvHD at DLI. All factors associated with GvHD in the univariate analysis with a *p* value <0.20 or considered as potentially relevant were included in the multivariate model. Then a backward stepwise selection procedure was used with a cutoff significance level of 0.05 for deleting factors. A separate analysis was performed for patients who received DLI as prophylaxis or for MC. All tests were two-sided. The type I error rate was fixed at 0.05 for determination of factors associated with time-to-event outcomes. R statistical software version 4.0.3 (R Core Team (2020) was used (R: A language and environment for statistical computing. R Foundation for Statistical Computing, Vienna, Austria. URL: https://www.R-project.org).

## Results

### Patients

Three-hundred and eighteen patients suffering from acute myeloid leukemia (AML, 78%) or acute lymphoblastic leukemia (ALL, 22%) with a median age of 47.5 years (range: 18.2–70.6, 49 were older than 60) were identified. They had received DLI in CHR for MC (*n* = 169, 53%), persisting or recurrent MRD (*n* = 23, 7%), or as prophylaxis (*n* = 126, 40%). From the latter subgroup, 89 patients had been reported earlier in another context [20] and were updated for the present analysis. Disease status at alloSCT was complete remission in 83% (first CR [CR1], 69%, second CR [CR2], 14%) and advanced disease in 17%. Before alloSCT, 58% had received in vivo TCD, 19% ex vivo TCD, 7% in vivo plus ex vivo, and 16% no TCD. Donors were MSD (64%) or MUD (36%). Further details are provided in Table 1.

**Table 1:** Characteristics of 318 patients receiving DLI in complete hematologic remission after allogeneic stem cell transplantation for acute leukemia.

Variable		Entire cohort		PreDLI for mixed chimerism		PreDLI for MRD/molecular relapse		ProDLI	
Number	*n* (% of the entire cohort)	318 (100%)		169 (53.1%)		23 (7.2%)		126 (39.6%)	
Follow-up after DLI (months)	Median [IQR]	84.0 [49.1–110.9]		81.1 (43.6–106.7)		72.7 (46.3–90.6)		93.8 (58.0–130.1)	
Patient age (years)	Median (min–max) [IQR]	47.5 (18.2–70.6) [37.5–56.3]		49.8 (18.5–69) [39.8–58.1]		37.8 (20.5–60.1) [26.5–48.3]		46.1 (18.2–70.6) [37.6–54.3]	
Year of alloSCT	Median (range)	2007 (2001–2010)		2007 (2001–2010)		2007 (2001–2010)		2005 (2001–2010)	
Patient sex	Male	178 (56%)		98 (58%)		13 (56.5%)		67 (53.2%)	
	Female	140 (44%)		71 (42%)		10 (43.5%)		59 (46.8%)	
Diagnosis	AML	249 (78.3%)		137 (81.1%)		16 (69.6%)		96 (76.2%)	
	ALL	69 (21.7%)		32 (18.9%)		7 (30.4%)		30 (23.8%)	
AML: cytogenetic subgroups [11]	Favorable	18 (7.8%)		10 (8.0%)		1 (6.7%)		7 (7.6%)	
	Intermediate	173 (74.6%)		99 (79.2%)		7 (46.7%)		67 (72.8%)	
	Adverse	41 (17.7%)		16 (12.8%)		7 (46.7%)		18 (19.6%)	
	Missing	17		12		1		4	
ALL: subtypes	Philadelphia negative B ALL	30 (52.6%)		14 (56.0%)		2 (28.6%)		14 (56.0%)	
	Philadelphia positive B ALL	19 (33.3%)		8 (32.0%)		5 (71.4%)		6 (24.0%)	
	T ALL	8 (14%)		3 (12.0%)		0		5 (20.0%)	
	Missing	12		7		0		5	
Time diagnosis to alloSCT (months)	Median (min–max) [IQR]	5.5 (1–185.1) [4.2–9.7]		5.6 (1.4–88.2) [4.4–10.1]		5.7 (1–64.7) [3.5–8.5]		5.1 (1.4–185.1) [4–8.6]	
Donor	Matched sibling donor	203 (63.8%)		113 (66.9%)		16 (69.6%)		74 (58.7%)	
	Unrelated donor	115 (36.2%)		56 (33.1%)		7 (30.4%)		52 (41.3%)	
Donor sex	Male	205 (64.9%)		108 (63.9%)		15 (65.2%)		82 (66.1%)	
	Female	111 (35.1%)		61 (36.1%)		8 (34.8%)		42 (33.9%)	
	Missing	2		0		0		2	
Female donor for male patient	Other	263 (83.2%)		140 (82.8%)		18 (78.3%)		105 (84.7%)	
	Female to male	53 (16.8%)		29 (17.2%)		5 (21.7%)		19 (15.3%)	
	Missing	2		0		0		2	
		Myeloablative	Reduced	Myeloablative	Reduced	Myeloablative	Reduced	Myeloablative	Reduced
Conditioning	Chemotherapy based	56 (33.7%)	107 (70.4%)	25 (33.8%)	82 (86.3%)	5 (35.7%)	6 (66.7%)	26 (33.3%)	19 (39.6%)
	TBI based	110 (66.3%)	45 (29.6%)	49 (66.2%)	13 (13.7%)	9 (64.3%)	3 (33.3%)	52 (66.7%)	29 (60.4%)
Status at alloSCT	CR1	219 (69.3%)		125 (74%)		11 (47.8%)		83 (66.9%)	
	CR2+	45 (14.2%)		29 (17.2%)		4 (17.4%)		12 (9.7%)	
	Advanced	52 (16.5%)		15 (8.9%)		8 (34.8%)		29 (23.4%)	
	Missing	2		0		0		2	
Stem cell source	Bone marrow	48 (15.1%)		19 (11.2%)		4 (17.4%)		25 (19.8%)	
	Peripheral blood	270 (84.9%)		150 (88.8%)		19 (82.6%)		101 (80.2%)	
T-cell depletion	No	51 (16.2%)		31 (18.5%)		11 (47.8%)		9 (7.3%)	
	In vivo TCD	182 (57.8%)		104 (61.9%)		11 (47.8%)		67 (54%)	
	Ex vivo TCD	61 (19.4%)		21 (12.5%)		1 (4.3%)		39 (31.5%)	
	Both	21 (6.7%)		12 (7.1%)		0 (0%)		9 (7.3%)	
	Missing	3		1		0		2	
*History of graft-versus-host disease after alloSCT (before DLI)*									
Acute GVHD grade II–IV	No	265 (83.9%)		137 (81.5%)		17 (73.9%)		111 (88.8%)	
	Yes	51 (16.1%)		31 (18.5%)		6 (26.1%)		14 (11.2%)	
	Missing	2		1		0		1	
Chronic GVHD	No	285 (90.2%)		145 (86.3%)		19 (82.6%)		121 (96.8%)	
	Yes	31 (9.8%)		23 (13.7%)		4 (17.4%)		4 (3.2%)	
	Missing	2		1		0		1	

*DLI* donor lymphocyte infusion, *IQR* interquartile range, *AML* acute myeloid leukemia, *ALL* acute lymphoblastic leukemia, *alloSCT* allogeneic stem cell transplantation, *TBI* total body irradiation, *CR* complete remission, *MRD* minimal residual disease, *TCD* T-cell depletion.

### DLI

The median interval between alloSCT and DLI1 was 176 days (interquartile range [IQR]: 132–260), it was slightly longer in preDLI (206 days in molecular relapse/persisting MRD, 190 in MC) than in proDLI (169 days). The T-cell dose at the first infusion (DLI1) showed considerable variability (IQR: 1 × 10^6^–1 × 10^7^ CD3+ cells/kg). Regardless of indication for DLI, the median dose was 1 × 10^6^/kg CD3+ cells/kg, 75% received less than 1 × 10^7^ CD3+ cells/kg. Patients received a median of 2 DLI (IQR: 1–3). Reasons to desist from further infusions were reaching the pre-planned number of infusions (57%), GvHD (18%), leukemia relapse (12%), reaching complete donor chimerism (5%), infection (1%), and other reasons (5%). Table 2 provides further details.

**Table 2:** Details of DLI given to 318 patients in complete hematologic remission.

		Entire cohort	PreDLI for mixed chimerism	PreDLI for MRD/molecular relapse	ProDLI
Number	*n* (% of the entire cohort)	318 (100%)	169 (53.1%)	23 (7.2%)	126 (39.6%)
Time alloSCT to first DLI	Median (min–max) [IQR]	176 (15–1145) [132–260]	190 (15–1145) [126–314]	206 (42–589) [122.5–397]	169 (30–606) [139.2–210.2]
CD3+ cell/kg at first DLI (×10^6^/kg)	Median (min–max) [IQR]	1 (0.1–70) [1–10]	1 (0.1–70) [1–3]	1 (0.1-70) [1–10]	1 (0.1–70) [0.5-10]
	Missing	33	18	1	14
Groups according to CD3+ dose at first DLI (×10^6^/kg)	<1	65 (22.8%)	29 (19.2%)	5 (22.7%)	31 (27.7%)
	1–5	128 (44.9%)	86 (57%)	8 (36.4%)	34 (30.4%)
	≥5	92 (32.3%)	36 (23.8%)	9 (40.9%)	47 (42%)
	Missing	33	18	1	14
Number of DLI infusion given	Median [IQR]	2 [1–3]	2 [1–3]	3 [2–3.5]	1 [1–3]
Time DLI1–2 if 2 DLI (days)	Median (min–max) [IQR]	36 (6–193) [32–48]	71.5 (7–521) [34–126.2]	42 (6–294) [33–62.5]	43.5 (12–2422) [32.2–73.5]
Time DLI2–3 if 3 DLI (days)	Median (min–max) [IQR]	42 (14–326) [34–63.5]	43.5 (8–516) [28–99.2]	47 (14–326) [38–89.5]	43 (29–459) [41–64]
Stopped of immunosuppressive medication before DLI	No	42 (14.2%)	29 (19.3%)	5 (22.7%)	8 (6.5%)
	Yes	253 (85.8%)	121 (80.7%)	17 (77.3%)	115 (93.5%)
	Missing	23	19	1	3
Use of immunosuppressive prophylaxis after DLI	No	280 (88.9%)	143 (89.4%)	22 (95.7%)	115 (92.7%)
	Yes	27 (8.6%)	17 (10.6%)	1 (4.3%)	9 (7.3%)
	Missing	11	9	0	2

*DLI* donor lymphocyte infusion, *MRD* minimal residual disease, *IQR* interquartile range, *alloSCT* allogeneic stem cell transplantation, *CR* complete remission.

### Response and outcome

Clinical response after preDLI was reported for 16 out of 21 informative patients (71%) with MRD/molecular relapse, based on decreasing MRD (*n* = 15), and improving peripheral blood counts without measured MRD (*n* = 1). Although responses were observed in both AML and ALL, no comparison could be performed due to low numbers in the different subgroups (Supplementary Table 1). Among recipients of preDLI for MC, improved donor chimerism was observed in 110/158 (70%) informative patients.

**Fig. 1:**
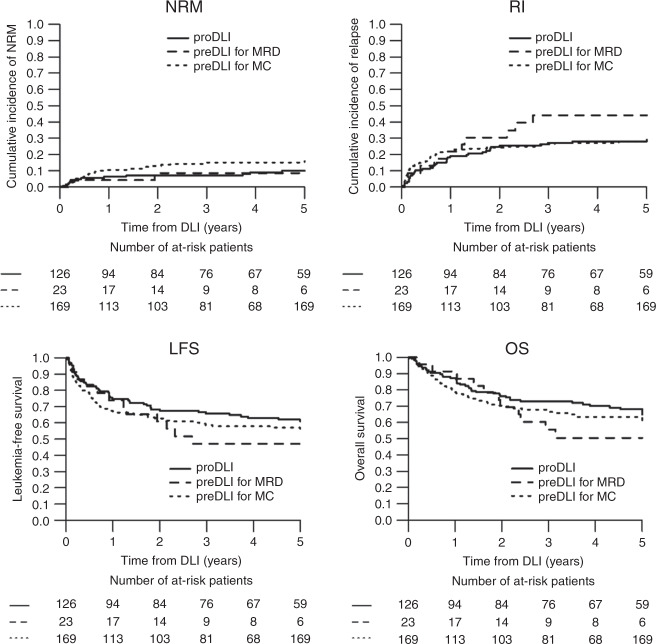
Outcome of 318 patients receiving donor lymphocyte infusion in complete hematological remission. NRM non-relapse mortality, CRI cumulative relapse incidence, LFS leukemia-free survival, OS overall survival. DLI was given as prophylaxis (red curves), as preemptive therapy for minimal residual disease (MRD) or molecular relapse (blue curves), or as preemptive therapy for mixed donor chimerism (green curves).

The median follow-up from DLI1 was 7.0 (IQR: 4.1–9.2) years. At 5 years, the rates of NRM, CRI, LFS, and OS of the entire cohort were 12.7% [9.2–16.7], 29.0% [24.2–34.3], 58.2% [52.7–63.7], and 64.3% [58.9–69.7], respectively. For the proDLI cohort, the 5-year NRM, CRI, LFS, and OS rates were 10%, 28%, 62%, and 68%. The respective results after preDLI for MRD were, 9%, 44%, 47%, and 51%; and for preDLI for MC they were 15%, 28%, 57%, and 63%. Overall, no relapses occurred beyond 3 years from DLI1 (Fig. 1). Among responders, the 5-year LFS and OS were 55% and 63% after preDLI for MRD/molecular relapse, and 68% and 76% after preDLI for MC, respectively. In contrast, the 5-year OS in non-responders was 37%.

Leukemia relapse was the most frequent cause of death, occurring in 62 patients (55% of all deaths, 19% of the entire cohort). Nineteen patients (17 % of all deaths, 6% of the entire cohort) died from GvHD (Supplementary Table 2). In a risk factor analysis for outcome after proDLI, no factor evaluable at time of DLI could be identified as being prognostic for outcome; there was a trend for better OS among patients with AML as compared to ALL. In contrast, prior transplantation in CR1 and a longer interval between SCT and first DLI were associated with better LFS and OS after preemptive DLI for MC (Supplementary Tables 3 and 4).

### GvHD induced by DLI

For the analysis of risk factors for DLI-induced GvHD and its influence on outcome, 70 patients had to be excluded due to clinical signs of GvHD at time of DLI (*n* = 12), prophylactic immunosuppression given after DLI (*n* = 42) or missing information on GvHD (*n* = 16). Accordingly, we selected 248 (47.7% receiving proDLI, 52.3%) receiving preDLI; patients who had received DLI in the absence of active GvHD, were off immunosuppressive medication by the day of DLI, and who also did not receive prophylactic immunosuppression after DLI. Outcome was comparable among patients included and excluded from this analysis (data not shown). Within the selected cohort, aGvHD grade I, II, III, and IV were reported in 25, 21, 9, and 9 patients. The cumulative incidence of aGvHD grade II–IV after DLI was 11.9% (95% CI: 8.2–16.3%), median day of onset was day +51 from DLI1 (IQR: 21–91). Five-year cumulative incidence of cGvHD was 30.7% (95% CI: 24.9–36.6%), with a median onset at day +135 (IQR: 89–237; Fig. 2). Taken together, the cumulative incidence of clinically relevant aGvHD or cGvHD was 33.7% (95% CI: 27.8–39.6%) at 5 years. In detail, 43.5% of GvHD events were observed after DLI1, 25.9%, 21.2%, and 9.4% after DLI2, DLI3, and DLI4, respectively. No differences were observed among patients receiving DLI preemptively or as pure prophylaxis.

**Fig. 2:**
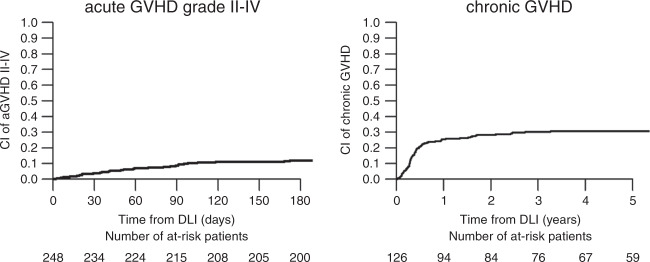
Cumulative incidence of acute graft-versus-host disease (aGvHD) grade II–IV and chronic GVHD after prophylactic or preemptive DLI. Only patients who had received DLI in the absence of active GvHD, were off immunosuppressive medication by the day of DLI, and did not receive prophylactic immunosuppression after DLI (*n* = 248) were selected.

With respect to outcome, neither aGvHD nor cGvHD was associated with decreased CRI. In contrast, NRM was 29% (25/85) and 2% (3/163) among patients who did or did not develop aGvHD grade II–IV or cGvHD after DLI, suggesting a clear association between GvHD and NRM. Accordingly, LFS (HR 2.20, 95% CI: 1.38–3.51, *p* = 0.001) and OS (HR 2.08, 95% CI: 1.34–3.22, *p* = 0.001) were significantly inferior among those patients who developed GvHD.

A detailed risk factor analysis for developing either aGvHD grade II–IV or cGvHD after DLI was performed. Due to increased risk of GvHD with >1 DLI, and since the reason to give >1 DLI could not be evaluated retrospectively, only patients receiving 1 single DLI were included in the model (*n* = 101). Age  > 60 years (*p* = 0.046), transplantation beyond CR1 (*p* = 0.003), a shorter interval from SCT to DLI (*p* = 0.018), and a history of aGvHD grade II–IV after SCT, but before DLI (*p* = 0.036) were associated with an increased risk for GvHD in multivariate analysis. In contrast, neither an unrelated donor, gender relationship between patient and donor, graft source or conditioning for SCT, or CD3+ cell count at DLI1, significantly influenced the occurrence of GvHD. See Table 3 for details.

**Table 3:** Risk factors for graft-versus-host disease after DLI in complete hematologic remission^a^.

*Univariate analysis*		*n*	GVHD after DLI [95% CI]
Diagnosis	AML	77	30.2% [20.2–40.8]
	ALL	24	29.2% [12.4–48.3]
	*p* value		0.84
Patient age	<Median	50	30.1% [17.9–43.2]
	≥Median	51	29.4% [17.5–42.3]
	*p* value		0.9
	18–35 years	19	47.4% [23.3–68.1]
	36–45 years	26	19.5% [6.8–37]
	46–60 years	41	24.4% [12.5–38.4]
	>60 years	15	40% [15.2–64]
	*p* value		0.17
Time alloSCT– DLI1	<Median	50	36.1% [22.9–49.5]
	≥Median	51	23.5% [12.9–36]
	*p* value		0.19
Status at alloSCT	CR1	72	23.6% [14.5–34]
	not CR1	27	50% [28.2–68.4]
	*p* value		0.015
Female donor for male patient	Yes	14	35.7% [12.2–60.4]
	No	86	29.2% [19.9–39.1]
	*p* value		0.49
Conditioning	Myeloablative	62	27.4% [16.9–39]
	Reduced intensity	39	33.3% [19–48.3]
	*p* value		0.27
Donor	Matched sibling	75	30.9% [20.7–41.6]
	Unrelated	26	26.9% [11.5–45]
	*p* value		0.95
Stem cell source at alloSCT	Bone marrow	18	44.4% [20.6–65.9]
	Peripheral blood	83	26.8% [17.6–36.7]
	*p* value		0.16
In vivo T-cell depletion	No in vivo TCD	49	26.6% [15.1–39.6]
	In vivo TCD	51	33.3% [20.8–46.4]
	*p* value		0.25
Ex vivo T-cell depletion	No ex vivo TCD	53	38.8% [25.2–52.2]
	Ex vivo TCD	48	20.8% [10.6–33.3]
	*p* value		0.027
aGVHD grade II–V before DLI	No aGVHD II–IV before DLI	88	27.5% [18.5–37.2]
	aGVHD II–IV before DLI	13	50% [18.9–74.9]
	Grade II	9	
	Grade III	4	
	*p* value		0.07
CD3 first DLI	CD3/Kg <median	46	40.4% [25.5–54.9]
	≥Median	47	23.4% [12.4–36.4]
			0.052
*Mulitvariate model*	GVHD after DLI		
	HR (95% CI)		*p* value
CR1 at HSCT	0.32 (0.15–0.68)		0.003
Time Tx-DLI1 > 184 days (median)	0.38 (0.17–0.84)		0.018
aGVHD grade II–IV before DLI	3.42 (1.09–10.75)		0.036
Age >60 years	2.55 (1.02–6.38)		0.046

*GVHD* graft-versus-host disease, *DLI* donor lymphocyte infusion, *CI* confidence interval, *CRI* cumulative relapse incidence, *CR* complete remission, *AML* acute myeloid leukemia, *ALL* acute lymphoblastic leukemia, *TCD* T-cell depletion.

^a^Only patients receiving 1 DLI (*n* = 101, 53% receiving prophylactic 47% receiving therapeutic DLI) were considered for the risk factor analysis.

## Discussion

This large retrospective registry study on more than 300 patients with a median follow-up of 7 years provides mature outcome data after prophylactic and preemptive infusion of unmodified DLI after alloSCT for AL. To evaluate the pure effect of donor cells, patients from the registry who had received any additional antileukemic therapy after SCT before or at time of DLI, such as TKI, HMA, or chemotherapy had been excluded from the analysis. Response was reported after preDLI given both for MRD/molecular relapse and for MC. Improved long-term outcome was observed among responders.

Concerning patients receiving proDLI, the lack of a control group precluded a firm statement on antileukemic efficiency. This question has been addressed in a matched-pair analysis including a subgroup of the patients reported and updated here, which had shown a significantly improved OS and LFS after proDLI in patients with high-risk AML, but not in standard-risk AML and ALL [20]. Nevertheless, 5-year LFS/OS rates of 62%/68% in the larger series analyzed here were encouraging, given the high-risk characteristics of the cohort, which included 1/3 of patients transplanted beyond CR1, and 1/3 having received ex vivo T-cell-depleted grafts. These data confirm results from earlier studies using proDLI after TCD for SCT [21], and in T-cell replete SCT in high-risk AML and MDS [22, 23]. Nevertheless, prospective trials in well-defined cohorts are warranted to define the role of proDLI.

With respect to efficacy of preDLI, earlier studies had reported improved chimerism and promising outcome in children and adults with AML, receiving preDLI for mixed donor chimerism [24–26]. Similarly, antileukemic effects of unstimulated [27, 28] or modified [29] preDLI triggered by MRD or molecular relapse have been suggested. In our study, clinical response to preDLI was observed in around 70% of patients both treated for MC and MRD/molecular relapse, although the data in the latter subgroup must be interpreted with caution due to low numbers and heterogenous measurement techniques [30]. Long-term OS from DLI was achieved among responders of both cohorts (55% after MRD triggered, 76% after MC-triggered preDLI), whereas OS was 37% only among non-responders. Unfortunately, no formal comparison among responders and non-responders could be performed due to missing information on the exact date of response. However, the differences at least suggest a clinical relevance of preDLI, supporting in a large series prior data from smaller studies. In a risk factor analysis, we identified SCT in CR1 and a longer interval from the date of transplant as favorable factor for both OS and LFS after preDLI for MC. While CR1 at transplant is a favorable factor for outcome in general, the longer interval might reflect a later occurrence of MC, possibly indicating less dynamic disease. Time between SCT and cellular intervention is a well-known risk factor also for therapeutic DLI and second SCT for hematological relapse.

GvHD was the most devastating complication of DLI in CHR, leading to an increase of NRM and inferior LFS/OS without influencing the risk of relapse. In fact, GvHD was the cause of death in 19, representing 6% of the entire cohort and 17% of all deaths. Hence, identification of risk factors for DLI-induced GvHD after DLI was of great interest. To avoid bias by sequential DLI, pre-existing GvHD, and concomitant immunosuppressive medication, we limited the risk factor analysis to patients receiving exactly one unmanipulated DLI in the absence of active GvHD and off immunosuppressive medication, and who also did not receive prophylactic immunosuppression after DLI. Not unexpectedly, a history of aGvHD grade II–IV after SCT was an independent risk factor for DLI-induced GvHD. Hence, both preDLI and proDLI should probably be used carefully or even avoided in patients who had suffered from severe aGvHD before. The same is true for patients above the age of 60, who also had an increased risk of DLI-induced GvHD and might be more vulnerable to organ damage caused by GvHD and consecutive immunosuppressive treatment. Furthermore, the time interval between SCT and DLI1 is critical for the safety of DLI. Early studies had revealed excessive rates of GvHD with application during the first 30–60 days after SCT [31], which is why a delay at least until day +120 after SCT was a prerequisite for proDLI application in another study [32]. Our data confirm the outstanding role of a shorter time interval between SCT and DLI1 as risk factor for the development of clinically relevant GvHD. This also represents a practical problem at least for using proDLI, since early relapse cannot be prevented by delayed DLI. Either combination with cytoreductive or immune-modulating drugs [33, 34] or modifications of classical DLI that can be applied earlier after transplantation [35, 36] might help to overcome this limitation. Later on, DLI can be repeated using escalating cell doses, based on the development of GvHD and clinical response [22].

Several limitations of our study need to be considered. First, as a typical drawback of a registry analysis, the reason why the patients described here had received DLI in CHR, whereas others did not, could not be evaluated retrospectively, implicating the risk of a selection bias. Second, both MRD and MC were measured locally by the reporting centers, using different methods and cutoffs [30]. This precluded the quantification of MC or MRD and reproducible cutoff values, e.g., for a level of chimerism justifying the use of DLI cannot be provided. Similarly, estimates of the extent and quality and hence the clinical relevance of the response to preDLI cannot be given. However, response data were based on the same method at each individual center, which is why the overall message in terms of response rates can be regarded as reliable. Third, although collecting data from one of the largest registries available, numbers were too small to perform meaningful subgroup analyses, e.g., between AML and ALL, or based on the method of TCD used for SCT. In particular, the role of prior ex vivo TCD might have been mitigated by low numbers and an association with patient’s age. Finally, CD3+ cell counts at the various DLIs varied considerably among patients and were missing in a considerable number, which is why the influence of cell dose on efficacy and the development of GvHD might be underestimated, whereas it was reported to be relevant in earlier studies [37, 38].

In summary, our data provide long-term data on outcome and safety of preemptive and proDLI after SCT for AL, and help to identify candidates for DLI in CHR without increased risk of severe GvHD. Nevertheless, due to high relapse rates, in particular among patients receiving preDLI for MRD, the data also underscore that unmanipulated DLI alone may be insufficient to reliably prevent post-transplant relapse in AL in many cases. Hence, the combined use of targeted or immune-modulating drugs and DLI+/– short-term immunosuppression (summarized in [39]) or the application of specifically educated T cells [40] might represent more effective ways to improve maintenance and preemptive therapy after SCT for high-risk AL.

## Supplementary information

Supplementary Table 1

Supplementary Table 2

Supplementary Table 3

Supplementary Table 4

Supplementary Table 5
