# Supplementary material for: Long-term results and GvHD after prophylactic and preemptive donor lymphocyte infusion after allogeneic stem cell transplantation for acute leukemia
Source: Bone Marrow Transplant. 2021 Nov 8;57(2):215–23. doi: 10.1038/s41409-021-01515-3 (PMC8821014; doi:10.1038/s41409-021-01515-3)
Supplement: Supplementary file 1 — Supplementary Table 1 [file 41409_2021_1515_MOESM1_ESM.pdf]

Supplementary table 1: Response to preemptive DLI by diagnose

AML

	Reason for DLI		Total
	MRD/molecular relapse	MC	
Response, n (%)	13 (81.3%)	92 (71.9%)	105
No response n (%)	3 (18.7%)	36 (28.1%)	39
missing	0	9	9
Total, n	16	137	153

ALL

	Reason for DLI		
	MRD/molecular relapse	MC	
Response, n (%)	3 (60%)	18 (60%)	21
No tesponse n (%)	2 (40%)	12 (40%)	14
missing	2	2	4
Total, n	7	32	39

Note: DLI, donor lympohocyte infusic Reason for DLI Total
