# Supplementary material for: Long-term results and GvHD after prophylactic and preemptive donor lymphocyte infusion after allogeneic stem cell transplantation for acute leukemia
Source: Bone Marrow Transplant. 2021 Nov 8;57(2):215–23. doi: 10.1038/s41409-021-01515-3 (PMC8821014; doi:10.1038/s41409-021-01515-3)
Supplement: Supplementary file 2 — Supplementary Table 2 [file 41409_2021_1515_MOESM2_ESM.pdf]

Supplementary table 2: Cause of death

	n (%)	MC	MRD	proDLI
N	119	66	11	42
Original disease	62 (55.4%)	33 (54.1%)	8 (72.73%)	21 (52.5%)
Secondary malignancy	1 (0.9%)	0 (0%)	0 (0%)	1 (2.5%)
GVHD	19 (17.0%)	10 (16.39%)	1 (9.09%)	8 (20%)
Infection	18 (16.1%)	9 (14.75%)	1 (9.09%)	8 (20%)
Interstitial pneumonia	4 (3.6%)	4 (6.56%)	0 (0%)	0 (0%)
Haemorrhage	3 (2.7%)	1 (1.64%)	1 (9.09%)	1 (2.5%)
VOD	2 (1.8%)	1 (1.64%)	0 (0%)	1 (2.5%)
Other transp related causes	3 (2.7%)	3 (4.92%)	0 (0%)	0 (0%)
Missing	7	5	0	2

Note: GVHD, Graft-versus-Host disease; VOD, veno-occlusive disease
