# Supplementary material for: Long-term results and GvHD after prophylactic and preemptive donor lymphocyte infusion after allogeneic stem cell transplantation for acute leukemia
Source: Bone Marrow Transplant. 2021 Nov 8;57(2):215–23. doi: 10.1038/s41409-021-01515-3 (PMC8821014; doi:10.1038/s41409-021-01515-3)
Supplement: Supplementary file 3 — Supplementary Table 3 [file 41409_2021_1515_MOESM3_ESM.pdf]

Supplementary table 3: Risk factor analysis for 5 year outcome afterprophylactic DLI (n=126)

Univariate analysis

		Relapse	NRM	LFS	OS
Patient age	age<median (46y)	32%[20.8-43.7]	6.6%[2.1-14.8]	61.5%[48.2-72.3]	69.5%[56.3-79.3]
	age>median	24%[14.2-35.2]	13.6%[6.2-23.7]	62.4%[48.9-73.3]	66.6%[53-77.1]
	P value	0.21	0.2	0.68	0.93
classes	(18,35]	40%[22.4-57]	6.7%[1.1-19.5]	53.3%[34.3-69.1]	63.3%[43.6-77.8]
	(35,45]	24.1%[10.4-40.9]	7.5%[1.2-21.7]	68.4%[47.9-82.2]	75.1%[54.6-87.4]
	(45,60]	27.6%[15.7-41]	14.2%[5.5-26.7]	58.2%[42.2-71.2]	63.5%[47-76.1]
	(60+	15.8%[3.7-35.6]	10.5%[1.7-29]	73.7%[47.9-88.1]	73.7%[47.9-88.1]
	P value	0.34	0.75	0.52	0.4
	(18,60]	30.2%[21.7-39.2]	9.9%[5-16.8]	59.8%[49.7-68.6]	67.1%[57-75.3]
	(60+	15.8%[3.7-35.6]	10.5%[1.7-29]	73.7%[47.9-88.1]	73.7%[47.9-88.1]
	P value	0.18	0.87	0.29	0.56
Time HCT DLI	<median (169d)	26.1%[15.8-37.6]	10.2%[4.1-19.6]	63.7%[50.2-74.5]	68.6%[55.2-78.8]
	>median	30%[19.1-41.5]	9.9%[4-19.1]	60.1%[46.9-71.1]	67.7%[54.5-77.9]
	P value	0.8	0.93	0.88	0.99
Year	<2005	22.7%[11.6-36.1]	15.9%[6.9-28.3]	61.4%[45.4-73.9]	63.6%[47.7-75.9]
	>=2005	31.1%[21.3-41.5]	6.4%[2.3-13.5]	62.4%[50.8-72.1]	71.2%[59.8-79.9]
	P value	0.41	0.1	0.85	0.79
Female to male combination	noFtoM	30.8%[22.1-39.8]	10%[5.1-16.9]	59.2%[49-68]	65.7%[55.5-74.1]
	FtoM	15.8%[3.7-35.6]	10.5%[1.6-29.1]	73.7%[47.9-88.1]	78.9%[53.2-91.5]
	P value	0.19	0.89	0.28	0.26
diagnosis	AML	25.4%[17.1-34.6]	9%[4.2-16.2]	65.6%[54.8-74.3]	72.9%[62.5-80.9]
	ALL	36.7%[19.8-53.8]	13.3%[4.1-28.2]	50%[31.3-66.1]	53.1%[34-69]
	P value	0.23	0.45	0.08	**0.049**
CR1	CR1	26.8%[17.7-36.8]	10.2%[4.7-18.3]	63%[51.4-72.5]	70%[58.6-78.9]
	not CR1	31.9%[18.2-46.5]	10.1%[3.1-21.9]	58%[41.4-71.5]	62.6%[45.7-75.6]
	P value	0.55	0.99	0.58	0.53
Intensity of conditioning	MAC	28.2%[18.7-38.5]	10.5%[4.9-18.7]	61.3%[49.4-71.1]	64.8%[52.9-74.3]

	RIC	28%[15.9-41.5]	8.9%[2.7-19.6]	63.1%[47.4-75.3]	74.2%[58.9-84.5]
	P value	0.75	0.8	0.66	0.78
TBI	CT	20.1%[9.8-33]	11.8%[4.2-23.8]	68%[51.9-79.7]	70.3%[54.2-81.6]
	TBI	32.2%[22.3-42.6]	8.9%[3.9-16.5]	58.8%[47.2-68.7]	67.2%[55.6-76.4]
	P value	0.14	0.59	0.3	0.6
Cell source	BM	24%[9.5-42.1]	12%[2.9-28.1]	64%[42.2-79.4]	64%[42.2-79.4]
	PB	29.1%[20.5-38.3]	9.4%[4.6-16.3]	61.5%[51-70.3]	69.3%[59-77.5]
	P value	0.54	0.71	0.69	0.9
Type of donor	MSD	31.2%[21-42]	6.9%[2.5-14.4]	61.8%[49.7-71.9]	71.1%[59.2-80.2]
	UD	23.1%[12.7-35.3]	15%[6.4-27.1]	61.9%[46.7-74]	63.6%[48.3-75.5]
	P value	0.28	0.16	0.88	0.54
	noFtoM	30.8%[22.1-39.8]	10%[5.1-16.9]	59.2%[49-68]	65.7%[55.5-74.1]
	FtoM	15.8%[3.7-35.6]	10.5%[1.6-29.1]	73.7%[47.9-88.1]	78.9%[53.2-91.5]
	P value	0.19	0.89	0.28	0.26
in vivo TCD	no invivo TCD	22.9%[12.2-35.7]	12.6%[5-23.7]	64.5%[49.3-76.2]	68.7%[53.5-79.8]
	invivo TCD	30.6%[20.5-41.3]	8.6%[3.4-16.7]	60.8%[48.6-71]	68.5%[56.3-78]
	P value	0.58	0.46	0.99	0.66
Ex vivo TCD	no exvivo TCD	29.7%[19.7-40.4]	10%[4.3-18.6]	60.3%[47.9-70.6]	68.6%[56.5-78]
	ex vivo TCD	26.5%[15.1-39.5]	10.3%[3.7-20.8]	63.2%[48.1-75]	67.3%[52.3-78.5]
	P value	0.98	0.95	0.93	0.75
Accute GvHD before DLI	no	27.3%[19.3-35.9]	9.4%[4.7-15.9]	63.4%[53.6-71.7]	68.6%[58.9-76.5]
	yes	28.6%[8.2-53.4]	15.2%[2.1-39.9]	56.2%[27.2-77.6]	69.8%[37.8-87.6]
	P value	0.98	0.54	0.74	0.81

Multivariate analysis

No factor significant; only a trend for a lower OS in ALL compared to AML (HR: 1.88, 95% CI: 0.99-3.58)

Note: DLI, donor lymphocyte infusion; IQR, interquartile range; AML, Acute myeloid leukemia; ALL, acute lymphoblastric leukemia; alloSCT: allogeneic stem cell transplantation, TBI, total body irradiation; CR: complete remission; MRD, minimal residual disease; TCD, T cell depletion; GvHD, Graft-versus-Host Disease
