# Supplementary material for: Long-term results and GvHD after prophylactic and preemptive donor lymphocyte infusion after allogeneic stem cell transplantation for acute leukemia
Source: Bone Marrow Transplant. 2021 Nov 8;57(2):215–23. doi: 10.1038/s41409-021-01515-3 (PMC8821014; doi:10.1038/s41409-021-01515-3)
Supplement: Supplementary file 4 — Supplementary Table 4 [file 41409_2021_1515_MOESM4_ESM.pdf]

Supplementary table 4: Risk factor analysis for 5 year outcome after preemptive DLI for mixed chimerism (n=169)

Univariate analysis

		LFS	OS
Patient age	age<=50 (median)	55.2%[43.7-65.2]	62.3%[50.8-71.8]
	age>50	59%[47.5-68.9]	64.4%[52.8-73.9]
	P value	0.8	0.93
classes	(18,35]	51.7%[32.5-67.9]	51.7%[32.5-67.9]
	(35,45]	54.1%[36.4-68.9]	58.4%[39.7-73.1]
	(45,60]	67.5%[55.4-76.9]	76.9%[65.4-85]
	(60+]	39.2%[20.9-57.1]	45.4%[25.9-62.9]
	P value	0.046	0.02
	(18,60]	60.7%[52-68.4]	67.1%[58.4-74.3]
	(60+]	39.2%[20.9-57.1]	45.4%[25.9-62.9]
	P value	**0.02**	**0.017**
Time BMT DLI	<190d (median)	46%[35-56.3]	54.3%[43-64.3]
	>190d	68%[56.5-77.1]	72.4%[61-81]
	P value	**0.002**	**0.004**
Year of HCT	YEAR<2007	56.9%[45.3-67]	63.2%[51.5-72.8]
	YEAR>=2007	56.6%[45.1-66.6]	63.9%[52.5-73.3]
	P value	0.98	0.83
Female to male combination	noFtoM	55.4%[46.6-63.4]	62.7%[53.9-70.3]
	FtoM	65.1%[44.8-79.5]	66.9%[45.5-81.4]
	P value	0.44	0.53
Diagnosis	AML	57.4%[48.5-65.4]	64.7%[55.8-72.2]
	ALL	56.1%[37.4-71.2]	58.2%[38.8-73.3]

	P value	0.98	0.68
CR1	CR1	67.4%[58.3-75]	71.8%[62.8-79]
	not CR1	26.7%[13.8-41.6]	39%[24.1-53.5]
	P value	**0.001**	**0.001**
Intensity of conditioning	MAC	63.4%[51.4-73.3]	68.8%[56.8-78]
	RIC	51.2%[40-61.3]	58.3%[46.9-68]
	P value	0.13	0.2
TBI	CT	51.2%[40.8-60.6]	59.7%[49.3-68.7]
	TBI	66%[52.8-76.4]	69%[55.8-79]
	P value	0.19	0.39
Cell source	BM	62.2%[36.4-80]	62.2%[36.4-80]
	PB	56.4%[47.9-64.1]	63.6%[55.1-70.9]
	P value	0.54	0.93
Type of donor	MSD	60.2%[50.4-68.7]	66.3%[56.5-74.3]
	UD	50.8%[36.9-63.2]	58%[43.8-69.9]
	P value	0.21	0.29
Female to male combination	noFtoM	55.4%[46.6-63.4]	62.7%[53.9-70.3]
	FtoM	65.1%[44.8-79.5]	66.9%[45.5-81.4]
	P value	0.44	0.53
in vivo TCD	no invivo TCD	61.5%[46.9-73.2]	67.1%[52.5-78.1]
	invivo TCD	54.3%[44.4-63.2]	61.4%[51.5-69.9]
	P value	0.65	0.82
ex vivo TCD	no exvivo TCD	56%[46.9-64.1]	63.1%[54-70.9]
	ex vivo TCD	60.6%[42-74.9]	63.6%[44.9-77.5]
	P value	0.48	0.82

acute GVHD II before DLI	no	56.6%[47.6-64.6]	63.9%[55-71.5]
	yes	57.5%[38.1-72.7]	60.2%[40.5-75.2]
	P value	1	0.97

Multivariate Analysis

	LFS		OS	
	HR (95% CI)	p value	HR (95% CI)	p value
Age >=60y	1.28 (0.72-2.3)	0.4	1.33 (0.73-2.44)	0.35
**AlloSCT Not in CR1**	**2.93 (1.77-4.84)**	**< 0.0001**	**2.88 (1.7-4.87)**	**< 0.0001**
**Time SCT to DLI>median (190d)**	**0.46 (0.29-0.74)**	**0.001**	**0.47 (0.29-0.78)**	**0.003**

Note: DLI, donor lymphocyte infusion; IQR, interquartile range; AML, Acute myeloid leukemia; ALL, acute lymphoblastric leukemia; alloSCT: allogeneic stem cell transplantation, TBI, total body irradiation; CR: complete remission; MRD, minimal residual disease; TCD, T cell depletion; GvHD, Graft-versus-Host Disease
