# Supplementary material for: Long-term results and GvHD after prophylactic and preemptive donor lymphocyte infusion after allogeneic stem cell transplantation for acute leukemia
Source: Bone Marrow Transplant. 2021 Nov 8;57(2):215–23. doi: 10.1038/s41409-021-01515-3 (PMC8821014; doi:10.1038/s41409-021-01515-3)
Supplement: Supplementary file 5 — Supplementary Table 5 [file 41409_2021_1515_MOESM5_ESM.pdf]

Supplementary table 5: Participating centers

EBMT CIC Center	number of patients included
237 Nijmegen [St Radboud]	38
203 Leiden [Univ H]	36
311 Wiesbaden [Kl Diagnostik]	28
295 Hannover [Medical Univ]	24
810 Freiburg [University]	22
726 Liege [University]	17
212 Stockholm [Univ H]	15
276 Newcastle-Upon-Tyne [Royal Victoria]	15
230 Marseille [Paoli Calmettes]	14
297 Frankfurt_am_Main [Goethe-Universität]	13
513 Munich [Kl Grosshadern]	11
780 Manchester [Christie]	10
725 St._Petersburg [Pavlov Med Univ]	7
248 Pescara [Osp Civile]	6
284 Birmingham [Heartlands H]	5
387 Birmingham [Queen Elizabeth]	5
587 Reggio_Calabria [Centro Trapianti]	5
152 Augsburg [Zentra Kl]	4
676 Vandoeuvre_Les_Nancy [H d`Enfants]	4
597 Brno [Univ H]	3
666 Villejuif [Gustave Roussy]	3
672 Strasbourg [H Hautepierre]	3
169 Ankara [Gazi Univ]	2
202 Basel [202]	2
228 Edinburgh [Western General]	2
304 Firenze [Careggi-Meyer]	2
659 Brest [C.H.R.U Brest]	2
693 Warsaw [Inst Haematology]	2
728 Madrid [Puerta de Hierro]	2
754 Tel-Hashomer [Univ Adults]	2
926 Montpellier [University]	2
138 Frankfurt_am_Main [Kl Goethe U]	1
145 Stuttgart [Robert Bosch Kh]	1
225 Turku [University]	1
345 Haifa [Rambam MCH]	1
501 Liverpool [Royal Univ H]	1
530 Greifswald [Ernst-Moritz-Arndt]	1
614 Hamburg [Univ H]	1
677 Katowice [Silesian Med Acad]	1
718 Pilsen [Charles Univ H]	1
825 Alessandria [SS Antonio e Biagio]	1
919 Antalya [Medical Park H]	1
996 Antwerp_Edegem [UZA]	1
**Total**	**318**
